# Survival of medial versus lateral unicompartmental knee arthroplasty: A meta-analysis

**DOI:** 10.1371/journal.pone.0228150

**Published:** 2020-01-24

**Authors:** Seung-Beom Han, Sang-Soo Lee, Kyoung-Ho Kim, Jung-Taek Im, Phil-Sun Park, Young-Soo Shin

**Affiliations:** 1 Department of Orthopedic Surgery, Korea University Anam Hospital, Korea University College of Medicine, Seoul, Republic of Korea; 2 Institute for Skeletal Aging & Orthopedic Surgery, Chuncheon Sacred Heart hospital, Hallym University School of Medicine, Chuncheon, Republic of Korea; 3 Department of Orthopedic surgery, Armed Forces Daejeon Hospital, Daejeon, Republic of Korea; 4 Department of Orthopedic Surgery, Veterans Health Service Medical Center, Seoul, Republic of Korea; 5 Department of Orthopedic Surgery, Chuncheon Sacred Heart hospital, Hallym University School of Medicine, Chuncheon, Republic of Korea; Cleveland Clinic, UNITED STATES

## Abstract

Many studies have found associations between unicompartmental knee arthroplasty (UKA) and implant survival, but controversy still exists regarding the relative survival of medial versus lateral UKA over mid-to long-term follow-up. The purpose of this study was to compare survival and clinical outcomes of medial and lateral UKAs. In this meta-analysis, we reviewed studies that assessed implant survival in patients who underwent medial or lateral UKA with short- to mid-term (<10years) or long-term (>10years) follow-up, and that used assessments, such as pain and function scores, to compare postoperative scores on knee outcome scales. A total of eight studies (33,999 knees with medial UKA and 2,853 with lateral UKA) met the inclusion criteria and was analyzed in detail. There were no significant differences between medial and lateral UKA in pain score (95% CI: -0.37 to 0.88; P = 0.42), function score (95% CI: -0.19 to 0.60; P = 0.31), short- to mid-term survival (medial, 32,083/33,483; lateral, 2,636/2,726; OR 0.98, 95% CI: 0.64 to 1.48;P = 0.91), or long-term survival (medial, 479/516; lateral, 110/127; OR 2.51, 95% CI:0.67 to 9.43; P = 0.17). In addition, both groups had substantial proportions of knees with short- to mid-term survival (95.6% by medial UKA and 94.6% by lateral UKA) and long-term survival (92.8% by medial UKA and 86.6% by lateral UKA). This meta-analysis found no significant differences in short- to mid-term and long-term survival of medial and lateral UKAs. Similarly, patients treated with medial UKA showed no difference in pain relief or functional improvement compared to patients treated with lateral UKA. These results suggest that both UKA techniques are viable treatment options for patients with unicompartmental knee osteoarthritis over long-term follow-up, although further high-quality studies are needed to address some remaining uncertainties regarding the clinical benefits of these procedures.

## Introduction

Unicompartmental knee arthroplasty (UKA) is a bone-preserving and ligament-sparing procedure that reliably restores normal kinematics for osteoarthritis (OA) limited either to the medial or lateral compartment of the knee. This procedure allows for faster recovery, shorter hospital stay, and greater postoperative satisfaction compared to total knee arthroplasty (TKA).[1, 2] In general, medial UKA is performed more often than lateral UKA because the medial compartment is associated with higher incidence of disease, including OA and osteonecrosis, compared to the lateral compartment. In addition, lateral UKA is technically more challenging than medial UKA because of anatomic and kinematic differences between the two compartments and implant design factors; these factors have subsequently led to high revision rates for lateral UKA, even though postoperative valgus alignment with overcorrection is one of the main failure modes of medial UKA.[3, 4, 5] These findings indicate that alignment errors may cause complications, such as change in knee kinematics, wear rate, or implant loosening. Many studies have investigated implant survival and clinical outcomes of patients who underwent medial or lateral UKA. These studies have illuminated postoperative outcomes and provided valuable information regarding the advantages and disadvantages of each procedure. However, the evidence is limited because few comparative studies have been conducted, and only one systematic review is available.[6] Furthermore, published comparative studies have not yet yielded consistent results.[7, 8, 9, 10] The current study made use of the existing evidence to conduct a pairwise meta-analysis examining the survival and clinical outcomes of medial and lateral UKAs in patients who underwent unicompartmental knee OA. We hypothesized that implant survival and clinical outcomes would be similar between medial and lateral UKAs at final follow-up.

## Materials and methods

This meta-analysis was conducted according to the guidelines of the preferred reporting items for systematic reviews and meta-analysis (PRISMA) statement (S1 PRISMA Checklist)

### Data and literature sources

Although the current study involved human participants, ethical approval or informed consent from the participants was not required because all the data were from previously published studies and were analyzed anonymously without any potential harm to participants. The comprehensive databases of MEDLINE (January 1, 1976 to March 31, 2019), EMBASE (January 1, 1985 to March 31, 2019), Web of Science (January 1, 1980 to March 31, 2019), SCOPUS (January 1, 1980 to March 31, 2019), and the Cochrane Library (January 1, 1987 to March 31, 2019), were searched for studies that compared pain and function scores and implant survival rates in patients treated with medial or lateral UKA with short- to midterm (<10 years) or long-term (>10 years) follow-up. There were no restrictions on language. Search terms used in the title, abstract, MeSH, and keywords fields were (‘knee’ [MeSH] OR ‘arthroplasty’ [MeSH] OR ‘knee prosthesis’ [MeSH] OR ‘survivorship’ [MeSH]) AND ‘unicompartmental knee arthroplasty’ [tiab] OR ‘UKA’ [tiab] OR ‘unicondylar knee replacement’ [tiab] OR ‘knee arthroplasty’ [tiab] OR ‘lateral’ [tiab] OR ‘medial’ [tiab] OR ‘revision’ [tiab] OR ‘treatment outcome’ [tiab] OR ‘survivorship’ [tiab] OR ‘survival rate’ [tiab] OR ‘clinical outcome’ [tiab]). After the initial electronic search, additional relevant articles and bibliographies from identified studies were hand searched through other sources, including abstracts from annual meetings of the American Academy of Orthopedic Surgeons (AAOS) and the Osteoarthritis Research Society International (OARSI). We also searched weekly downloads of “Arthroplasty” articles in 6 journals (American Journal of Orthopedics; Archives of Orthopedic and Trauma Surgery; Journal of Arthroplasty; Journal of Bone and Joint Surgery American volume; Journal of Bone and Joint Surgery British volume; Orthopedics). The search was performed independently by two reviewers.

### Study selection

Two reviewers independently selected relevant studies for full review by searching through titles and abstracts. The full text copy of each article was reviewed if the abstract did not provide enough data to make a decision. Studies were included in the meta-analysis if they (1) assessed implant survival rates and clinical outcomes of patients who underwent medial or lateral UKA; (2) had follow-up duration of 3 years or longer; (3) simultaneously reported direct comparisons of medial and lateral UKA in studies published after 2000, to avoid out-of-date prosthetic models; (4) included basic data on at least one of the following three parameters: postoperative pain scores, function scores, or survival rates; (5) reported the number of subjects in each group and the means and standard deviations for the three parameters, and (6) used adequate statistical methods to compare parameters between groups. Postoperative scores on knee outcome scales were the Knee Society Score (KSS), International Knee Society (IKS) score, Western Ontario and McMaster Universities Osteoarthritis Index (WOMAC), and modified Hospital for Special Surgery (HSS) score. When an article offered data on multiple knee outcome scales, the Western Ontario and McMaster Universities Arthritis Index (WOMAC) was adopted to evaluate clinical outcomes. If the WOMAC was not reported, other relevant measurement scales such as KSS, IKS, HSS were applied instead. Studies were excluded if they (1) had missing or inadequate outcome data, such as standard deviations or ranges of values; (2) were case reports, expert opinions, reviews, commentaries, or editorials; (3) were abstracts only; (4) focused on animal in vivo or human in vitro work.

### Data extraction and assessment of methodological quality

Two reviewers independently recorded data from each study using a predefined data extraction form and resolved any differences by discussion. Recorded variables were those associated with surgical outcomes, such as postoperative pain, functional outcome, and survival rates, for patients with either medial or lateral UKA. Sample size and the means and standard deviations of surgical outcomes in each group were also recorded. Two reviewers independently assessed the methodological quality of the studies. For the Newcastle-Ottawa Scale, as recommended by the Cochrane Non-Randomized Studies Methods Working Group, we assessed studies based on three criteria: selection of the study groups, comparability of the groups, and ascertainment of either the exposure or the outcome of interest for case-control and cohort studies. The maximum score observed was 9 points, and total scores lower than 4 points were considered low in quality. Two reviewers resolved all differences by discussion, and their decisions were subsequently reviewed by a third investigator.

### Data synthesis and analysis

The main outcomes of the meta-analysis were proportions of cases that compared short-to mid-term (<10 years) and long-term (>10 years) survival between medial and lateral UKA. However, standardized mean difference (SMD) was calculated for overall functional outcome and postoperative pain because several different measurement tools, including KSS, IKS, WOMAC, and modified HSS, were used to measure the same outcome. For all comparisons, odds ratios (ORs) and 95% confidence intervals (CIs) were calculated for binary outcomes, while SMDs and 95% CIs were calculated for continuous outcomes. Comparable scores from different patient-reported pain and functional outcome instruments were combined as presented on a 100-point scale, where 0 indicates the worst pain imaginable and 100 indicates the absence of pain. When standard deviations (SDs) were not included in the original studies, they were calculated from the CIs or P values. Heterogeneity was determined by estimating the proportion of between-study inconsistencies due to actual differences between studies, rather than differences due to random error or chance. We assumed the presence of heterogeneity a priori and used the random-effects model in all pooled analyses. I^2^ statistics with a value less than 40% represent low heterogeneity, and a value of 75% or more indicates high heterogeneity.[11] When statistical heterogeneity was substantial, we conducted meta-regression to identify potential sources of bias such as study type and sample size. The age of the study subjects was also considered. All statistical analyses were performed with RevMan version 5.3 software and Stata version 14.2 static software. Subgroup analyses based on differences in follow-up period were performed for survival rates to explore a potential source of heterogeneity. As a result, two subgroups were created in each group: short-to mid-term (<10 years) and long-term (>10 years) survival rates. To detect the effect of individual studies on the pooled effect, sensitivity analysis was performed; five studies [12, 13, 14, 15, 16] with an all-polyethylene tibial component were included, and other studies [7, 12, 13, 14, 17] with a mobile bearing were included. Pooling of data was feasible for three outcomes of interest: survival rates, pain scores, and functional scores.

## Results

### Study identification, study characteristics, patient populations, quality assessment, and publication bias of included studies

**Fig 1:**
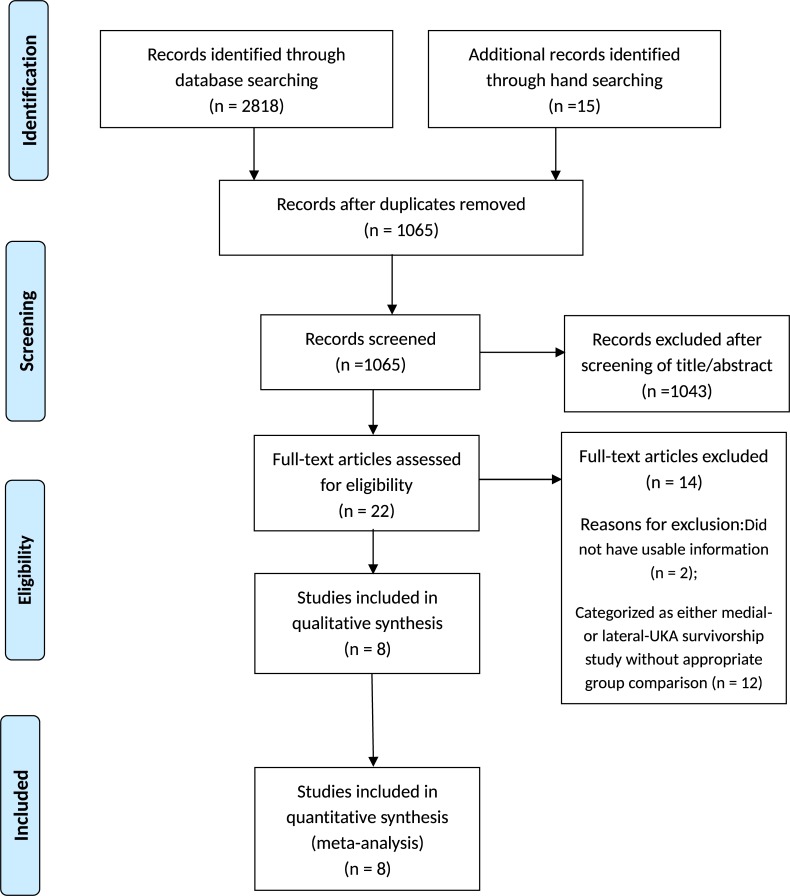
A flow diagram of preferred reporting items for systemic reviews and meta-analyses (PRISMA).

Details on study identification, inclusion, and exclusion are summarized in Fig 1. This process eventually resulted in eight studies in the final meta-analysis.[7, 12–18] An electronic search yielded 614 studies in PubMed (MEDLINE), 724 in EMBASE, 700 in Web of Science, 745 in SCOPUS, and 35 in the Cochrane Library. Fifteen additional publications were identified through manual searching. The eight studies we examined comprised 33,999 subjects with medial UKA and 2,853 subjects with lateral UKA that reported clinical outcomes, specific clinical scores, or survival rates. All eight studies compared parameters measured by retrospective chart review. Four studies compared groups according to short- to mid-term survival, four compared groups according to long-term survival, four compared groups according to pain score, and three compared groups according to functional score (Table 1). The scores for each study in the different groups were WOMAC [17], KSS [7,14], IKS [15], and HSS [7]. The quality of the eight studies is summarized in Table 1. Inter-rater reliability (k values) for all items of the Newcastle-Ottawa Scale ranged from 0.76 to 0.89, suggesting more than substantial agreement between the two investigators. Publication bias could not be assessed in these trials. Tests for funnel plot asymmetry are typically performed only when at least 10 studies are included in the meta-analysis. As our analysis included only eight studies, tests for asymmetry were not performed because these tests would not be able to differentiate asymmetry from chance.

**Table 1:** Summary of patient characteristics of the included studies.

Study	Year	Study type	Mean age (years)		Sample size (M/F)		Prosthesis properties Med/ Lat (tibial component)	Insert design	Follow-up (years)	Quality score	Measured parameters
					Med	Lat					
			Med	Lat					Med/ Lat		
Heyse et al.[13]	2011	RCS	53.7	53.7	173(NA)	50(NA)	Genesis/ Genesis (AP + MB)	fixed+ mobile	Mean 10.8/ Mean 10.0	NOS 8	LSR, PS, FS
Lustig et al.[14]	2008	RCS	72.2	72.2	84(NA)	60(NA)	Tonier/ Tonier (AP)	fixed	Mean 5.2/ Mean 5.2	NOS 8	SMRS, PS, FS
O'Rourke et al.[15]	2005	RCS	61.9	61.9	122(NA)	14(NA)	Marmor/ Marmor (AP)	fixed	Min 21.0/ Min 21.0	NOS 8	LSR
John et al.[17]	2011	RCS	66.5	66.5	76(NA)	18(NA)	Miller-Galante/ Miller-Galante (MB)	fixed	Mean 10.8/ Mean 10.8	NOS 7	LSR
Liebs et al.[16]^5^	2013	RCS	73.6	73.6	430(NA)	128(NA)	NA/ NA (MB)	mobile	Mean 6.0/ Mean 6.0	NOS 7	SMSR, PS, FS
Argenson et al.[7]	2008	OCS	68.0	61.0	145(NA)	40(15/24)	Miller-Galante / Marmor, Alpina, Miller-Galante, Zimmer (MB)	fixed + mobile	Mean 12.6/ Mean 12.6	NOS 7	LSR, PS
Gioe et al.[12]	2003	RCS	67.3	67.3	474(NA)	42(NA)	Osteonics, Kirschner/ Osteonics, Kirschner (AP + MB)	fixed + mobile	Mean 3.6/ Mean 3.6	NOS 7	SMSR
Baker et al.[11]	2012	RCS	64.6	63.1	30,795(16,223/14,572)	2,052(900/1,152)	Oxford, AMC Uniglide, Sled, UC-Plus/ Oxford, AMC Uniglide, Sled, UC-Plus (AP + MB + Modular)	fixed + mobile	Mean 6.5/ Mean 6.5	NOS 8	SMSR

Abbreviations: RCS, retrospective comparative study; OCS, observational case study; Med, medial; Lat, lateral; M, male; AP, all-polyethylene; MB, metal-backed; Min, minimum; SMSR, short- to midterm survival rate; LSR, long-term survival rate; PS, pain score; FS, functional score; NA, not available

### Survival rates

**Fig 2:**
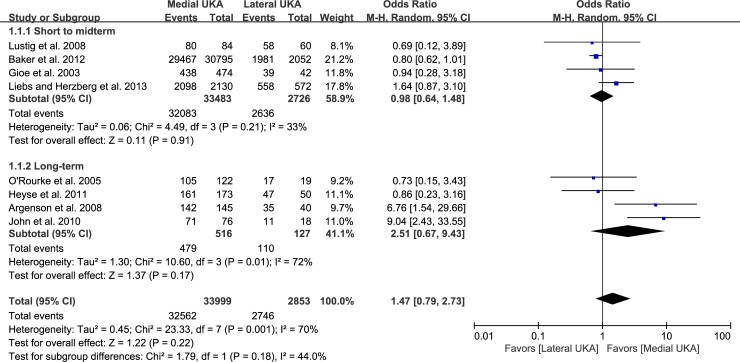
Results of aggregate analysis for comparison of short- to mid-term and long-term implant survival rates between patients with medial and lateral UKAs.

Eight studies compared survival rates between groups (medial UKA, 32,562/33,999; lateral UKA, 2,746/2,853; OR 1.47, 95% CI: 0.79 to 2.73; P = 0.22; I^2^ = 70%, Fig 2). Four studies were assigned to the short- and mid-term (<10 years) subgroup, and four studies were assigned to the long-term (>10 years) subgroup. For the short- and mid-term (<10 years) subgroup, the lateral UKA group had a higher survival rate than the medial UKA group, but this difference was not significant (medial UKA, 32,083/33,483; lateral UKA, 2,636/2,726; OR 0.98, 95% CI: 0.64 to 1.48; P = 0.91; I^2^ = 33%, Fig 2). Likewise, for the long-term (>10 years) subgroup, the medial UKA group had a higher survival rate than the lateral UKA group, but this difference was not significant (medial UKA, 479/516; lateral UKA, 110/127; OR 2.51, 95% CI: 0.67 to 9.43; P = 0.17; I^2^ = 72%, Fig 2). Both groups had substantial proportions of knees exhibiting short- to mid-term survival (95.6% by the medial UKA and 94.6% by the lateral UKA) and long-term survival (92.8% by the medial UKA and 86.6% by the lateral UKA). The sensitivity analysis found no significant differences compared to the original analysis, indicating that the findings were robust to decisions made in the data collection process (Table 2).

**Table 2:** Sensitivity analysis.

Study	Parameter	Before exclusion	After exclusion	Statistical significance
All-poly	SR	OR = 1.47, 95% CI = 0.79 to 2.73, Z = 1.22, P = 0.22	OR = 2.45, 95% CI = 0.88 to 6.83, Z = 1.72, P = 0.09	No difference
	PS	SMD = 0.03, 95% CI = -0.66, 0.71, Z = 0.07, P = 0.94	SMD = 0.37, 95% CI = -1.25, 1.99, Z = 0.45, P = 0.65	No difference
	FS	SMD = -0.08, 95% CI = -0.56, 0.40, Z = 0.33, P = 0.74	SMD = -0.40, 95% CI = -0.60, -0.20, Z = 3.93, P< 0.001	Difference
Mobile bearing	SR	OR = 1.47, 95% CI = 0.79 to 2.73, Z = 1.22, P = 0.22	OR = 2.50, 95% CI = 0.63 to 9.95, Z = 1.30, P = 0.19	No difference
	PS	SMD = 0.03, 95% CI = -0.66, 0.71, Z = 0.07, P = 0.94	SMD = 0.35, 95% CI = -1.32, 2.02, Z = 0.41, P = 0.68	No difference
	FS	SMD = -0.08, 95% CI = -0.56, 0.40, Z = 0.33, P = 0.74	SMD = 0.43, 95% CI = 0.09, 0.76, Z = 2.50, P = 0.01	Difference

SR, survival rate; PS, pain score; FS, function score; OR, Odds ratio;CI, confidence interval; SMD, standardized mean difference

### Clinical outcomes

**Fig 3:**
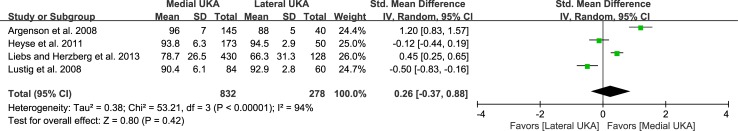
Results of aggregate analysis for comparison of pain scores between patients with medial and lateral UKAs.

**Fig 4:**
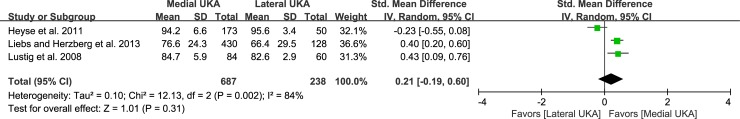
Results of aggregate analysis for comparison of function scores between patients with medial and lateral UKAs.

Of the 8 studies, 4 compared pain between patients with medial UKA (n = 832) and lateral UKA (n = 278). The pooled data showed that the standardized mean pain was 0.26 points (95% CI: -0.37 to 0.88 points; P = 0.42; I^2^ = 94%, Fig 3), with no significant difference between groups. The sensitivity analysis found no significant differences compared to the original analysis (Table 2). Three studies compared function between 687 subjects treated with medial UKA and 238 subjects treated with lateral UKA. The pooled data showed a standardized mean function of 0.21 points (95% CI: -0.19 to 0.60 points; P = 0.31; I^2^ = 84%, Fig 4), with no significant difference between groups. However, the results of sensitivity analysis with all-polyethylene and mobile bearing trials precluded showed significantly different SMD and 95% CI for the above effects compared with those of the original analysis (Table 2).

### Meta-regression analysis

The results of the meta-regression analysis are summarized in Table 3. For survival rates of the medial UKA group, age (P = 0.274), sample size (P = 0.935), and study type (P = 0.331) were not significant sources of heterogeneity. Similarly, age (P = 0.769), sample size (P = 0.211), and study type (P = 0.289) were not significant sources of heterogeneity for survival of the lateral UKA group.

**Table 3:** Meta-regression analyses of potential sources and difference in survival rate for medial or lateral UKA.

Variable	Coefficient	Standard error	P value	95% confidence interval
**Survival rate (medial UKA)**				
Age, mean, year (≤65 or ≥65)	-0.031	0.025	0.274	-0.093 to 0.032
Sample size, n (≤100 or ≥100)	-0.003	0.035	0.935	-0.088 to 0.082
Study type (RCS or Others)	0.038	0.036	0.331	-0.050 to 0.125
**Survival rate (lateral UKA)**				
Age, mean, year (≤65 or ≥65)	-0.012	0.038	0.769	-0.105 to 0.082
Sample size, n (≤100 or ≥100)	-0.034	0.024	0.211	-0.093 to 0.025
Study type (RCS or Others)	-0.092	0.079	0.289	-0.286 to 0.102

UKA, unicompartmental knee arthroplasty; RCS; retrospective comparative study

## Discussion

This pairwise meta-analysis analyzed 8 studies comprising 33,999 subjects treated with medial UKA and 2,853 subjects treated with lateral UKA. The results indicated that short- to mid-term and long-term survival did not differ significantly between medial and lateral UKA. In addition, both groups had substantial proportions of knees exhibiting short- to mid-term survival (95.6% by medial UKA and 94.6% by lateral UKA) and long-term survival (92.8% by medial UKA and 86.6% by lateral UKA). Furthermore, no significant difference was observed between the two treatment options with respect to pain relief or functional improvement.

It remains controversial whether medial or lateral UKA is superior in terms of short-to mid-term and long-term survival. In a previous follow-up study of unicompartmental implants, lateral UKA had a revision rate almost 10times higher than medial UKA.[14] In contrast, several studies have shown no significant difference in survival between medial and lateral UKA. There have even been studies in which lateral UKA had better survival.[5, 6, 15] Hypothetically, medial UKA should lead to longer survival and fewer failures than lateral UKA. There are two possible explanations for this: first, the surgical technique is much more demanding in lateral UKA. Even though the prevalence of isolated lateral compartment OA has been reported to range from 5% to 10%, lateral UKA comprises only 1% of knee arthroplasties annually.[10, 19] This trend can be explained by multiple factors, including the complex nature of the operation, surgeons’ lack of familiarity with it, and limited understanding of lateral unicompartmental OA. These possibilities are supported by the results of a study that found that the kinematics of the lateral compartment differ significantly from those of the medial side because internal tibial rotation during knee flexion increases posterior lateral condylar translation.[20] As a result, surgeons have narrow indications for lateral UKA and are thus reluctant to offer it, leading to high revision rates when this uncommon procedure is performed.[19] Second, many more studies have focused on development of implant materials and design in medial UKA compared to lateral UKA. Improvements in implant materials and design in medial UKA have allowed for more accurate alignment and positioning, which have markedly improved survival and functional outcomes.[21–24] The results of recent studies have shownthat newly developed lateral UKA systems are shaped appropriately to allow for sliding, rolling, and distracting forces, lowering the rate of dislocations in lateral UKA;[16, 25] whereas newly developed implant material and design have not improved survival in medial UKA. Furthermore, all UKA implants have been developed recently, and there is no single uniform implant. This lack of uniformity can lead to much lower survival with medial UKA than expected. This possibility is supported by the results of our study, as survival did not differ significantly between medial and lateral UKA, regardless of length of follow up. Our findings are similar to those of an earlier study in which survival for medial UKA was 94.1% at 10 years and 85.1% at 15 years compared with 91.8% at 10 and 15 years for lateral UKA.[14]

Similar to the results for survival, we observed no significant differences between medial and lateral UKAs in terms of postoperative pain and function scores. However, clinical results have varied across studies. One study that evaluated all health-related quality of life (HRQoL) results of 558 patients who underwent mobile-bearing UKA showed that patients treated with medial UKA had better outcomes than those treated with lateral UKA.[17] In contrast, another study that compared clinical outcomes between medial and lateral UKAs with a minimum follow-up of 2 years found no significant difference between the two types of partial knee arthroplasty with respect to WOMAC score.[26] It is possible that different insert designs and tibial components are important parameters for clinical outcomes after the two surgical procedures.[25–27] Our study showed that mobile-bearing inserts and all-polyethylene tibial components had an impact on functional scores but not pain scores in sensitivity analysis. However, the magnitude of observed difference between the two techniques was quite small and probably smaller than the minimum clinically important difference. Nevertheless, further research is needed to evaluate whether differences in HRQoL or WOMAC score are attributable to the procedure or to the disease.

This study had several limitations. All eight studies were observational, resulting in some inherent heterogeneity due to uncontrolled bias even though the studies had high quality scores. Another limitation involved the pooling of very heterogeneous data (different tibial components and insert designs used to determine the outcomes and variability in functional and pain scores), which were reflected by the I^2^ values of the various analyses. However, we did use a random effects model, sensitivity analysis, and meta-regression analysis to incorporate heterogeneous outcomes. Nonetheless, this heterogeneity should be considered when interpreting our findings. Lastly, more detailed prosthesis factors including brand may represent sources of unmeasured confounding, which is not feasible in this setting. Also, we could not know whether the leading causes for revision after UKAs including the progression of arthritis to a contralateral compartment, aseptic loosening explained by the smaller contact area between the implant and bone compared with TKAs, and the fact that patients undergoing UKAs are young and active, thus, their expectations are high, and the results of UKAs in these patients may be disappointing can cause directly patient death because the primary end point of our study was not mortality or the cause of death but the risk of revision surgery.

## Conclusions

This meta-analysis found that 95.6% of medial UKA and 94.6% of lateral UKA survived over a short- to mid-term follow up (<10 years), while 92.8% of medial UKA and 86.6% of lateral UKA survived over along-term follow up (>10 years). In addition, no significant differences were observed between the two treatment options with respect to survival rate, pain relief, and functional improvement. Thus, both UKA techniques appear to be viable treatment options for patients with unicompartmental knee OA over a long-term follow-up, although further high-quality studies are needed to address remaining uncertainties regarding the clinical benefits of these procedures.

## Supporting information

S1 PRISMA Checklist (DOC)

S1 Appendix (DOCX)
