# Supplementary material for: Survival of medial versus lateral unicompartmental knee arthroplasty: A meta-analysis
Source: PLoS One. 2020 Jan 24;15(1):e0228150. doi: 10.1371/journal.pone.0228150 (PMC6980580; doi:10.1371/journal.pone.0228150)
Supplement: S1 Appendix — (DOCX) [file pone.0228150.s002.docx]

Search Protocol

| **No.** | **MEDLINE** | **Results** |
| --- | --- | --- |
| #1 | "unicompartmental knee arthroplasty"[tiab] OR "unicompartmental arthroplasty"[tiab] OR "Unicondylar knee arthroplasty"[tiab] OR UKA[tiab] OR "Unicondylar Knee Replacement"[tiab] OR "Unicompartmental Knee Replacement"[tiab] OR "unicompartmental replacement"[tiab] OR "unicompartmental prostheses"[tiab] OR "unicompartmental prosthesis"[tiab] OR "unicondylar replacement"[tiab] | 1,798 |
| #2 | ("Arthroplasty, Replacement, Knee"[Mesh]) OR "Knee Prosthesis"[Mesh] | 26,448 |
| #3 | "knee arthroplasty"[tiab] OR "knee arthroplasties"[tiab] OR "knee replacement"[tiab] OR "knee prosthesis"[tiab] OR "knee prostheses"[tiab] | 27,983 |
| #4 | #2 OR #3 | 33,861 |
| #5 | (unicompartmental[ti]) OR (Unicondylar[ti]) | 1,544 |
| #6 | #4 AND #5 | 1,458 |
| #7 | #1 OR #6 | 1,928 |
| #8 | Lateral[tiab] OR laterally[tiab] OR medial[tiab] OR medially[tiab] | 374,278 |
| #9 | "Survivorship"[Mesh] OR "Survival Rate"[Mesh] OR "Treatment Outcome"[Mesh] | 1,072,810 |
| #10 | surviv*[tiab] OR Revision[tiab] OR outcome[tiab] | 1,855,761 |
| #11 | #9 OR #10 | 2,517,241 |
| #12 | #7 AND #8 AND #11 | 614 |

| **No.** | EMBASE | **Results** |
| --- | --- | --- |
| #1 | unicompartmental AND ('knee'/exp OR knee) AND ('arthroplasty'/exp OR arthroplasty) | 361 |
| #2 | unicompartmental knee arthroplasty':ab,ti OR 'unicompartmental arthroplasty':ab,ti OR 'unicondylar knee arthroplasty':ab,ti OR uka:ab,ti OR 'unicondylar knee replacement':ab,ti OR 'unicompartmental knee replacement':ab,ti OR 'unicompartmental replacement':ab,ti OR 'unicompartmental prostheses':ab,ti OR 'unicompartmental prosthesis':ab,ti OR 'unicondylar replacement':ab,ti | 2,032 |
| #3 | ((unicompartmental OR unicondylar) NEAR/2 (arthroplast* OR prosthes* OR replacement)):ab,ti | 2,073 |
| #4 | knee replacement'/exp OR 'knee arthroplasty'/exp OR 'knee prosthesis'/exp | 44,833 |
| #5 | knee arthroplast*':ab,ti OR 'knee replacement':ab,ti OR 'knee prosthes*':ab,ti | 34,142 |
| #6 | #4 OR #5 | 47,516 |
| #7 | unicompartmental:ab,ti OR unicondylar:ab,ti | 2,683 |
| #8 | #6 AND #7 | 2,326 |
| #9 | #1 OR #2 OR #3 OR #8 | 2,465 |
| #10 | lateral:ab,ti OR laterally:ab,ti OR medial:ab,ti OR medially:ab,ti | 461,174 |
| #11 | survivor'/exp OR 'survivorship'/exp OR 'survival rate'/exp OR 'treatment outcome'/exp OR 'clinical outcome'/exp | 1,722,262 |
| #12 | surviv*:ab,ti OR Revision:ab,ti OR outcome:ab,ti | 2,600,218 |
| #13 | #11 OR #12 | 3,586,456 |
| #14 | #9 AND #10 AND #13 | 724 |

| **No.** | Web of Science | **Results** |
| --- | --- | --- |
| #1 | TS=('unicompartmental knee arthroplasty' OR 'unicompartmental arthroplasty' OR 'Unicondylar knee arthroplasty' OR UKA OR 'Unicondylar Knee Replacement' OR 'Unicompartmental Knee Replacement' OR 'unicompartmental replacement' OR 'unicompartmental prostheses' OR 'unicompartmental prosthesis' OR 'unicondylar replacement') | 1,973 |
| #2 | TS=((unicompartmental OR unicondylar) NEAR/2 (arthroplast* OR prosthes* OR replacement)) | 1,723 |
| #3 | TS=('knee arthroplast*' OR 'knee replacement' OR 'knee prosthes*') | 43,053 |
| #4 | TI=(unicompartmental OR Unicondylar) | 1,372 |
| #5 | #3 AND #4 | 1,279 |
| #6 | #1 OR #2 OR #5 | 1,987 |
| #7 | TS=(lateral OR laterally OR medial OR medially) | 496,795 |
| #8 | TS=(surviv* OR Revision OR outcome) | 2,929,444 |
| #9 | #6 AND #7 AND #8 | 700 |

| **No.** | SCOPUS | **Results** |
| --- | --- | --- |
| #1 | TITLE-ABS-KEY("unicompartmental knee arthroplasty" OR "unicompartmental arthroplasty" OR "Unicondylar knee arthroplasty" OR UKA OR "Unicondylar Knee Replacement" OR "Unicompartmental Knee Replacement" OR "unicompartmental replacement" OR "unicompartmental prostheses" OR "unicompartmental prosthesis" OR "unicondylar replacement") | 2,334 |
| #2 | TITLE-ABS-KEY("knee arthroplast*" OR "knee replacement" OR "knee prosthes*") | 45,596 |
| #3 | TITLE(unicompartmental OR Unicondylar) | 1,831 |
| #4 | #2 AND #3 | 1,725 |
| #5 | #1 OR #4 | 2,460 |
| #6 | TITLE-ABS-KEY(lateral OR laterally OR medial OR medially) | 707,717 |
| #7 | TITLE-ABS-KEY(surviv* OR Revision OR outcome) | 4,610,928 |
| #8 | #5 AND #6 AND #7 | 745 |

| **No.** | **Cochrane** | **Results** |
| --- | --- | --- |
| #1 | ("unicompartmental knee arthroplasty"):ti,ab,kw OR ("unicompartmental arthroplasty"):ti,ab,kw OR ("Unicondylar knee arthroplasty"):ti,ab,kw OR (UKA):ti,ab,kw OR ("Unicondylar Knee Replacement"):ti,ab,kw | 103 |
| #2 | ((unicompartmental OR unicondylar) NEAR/2 (arthroplast* OR prosthes* OR replacement)):ti,ab,kw | 121 |
| #3 | MeSH descriptor: [Arthroplasty, Replacement, Knee] explode all trees | 2,210 |
| #4 | MeSH descriptor: [Knee Prosthesis] explode all trees | 0 |
| #5 | ("knee arthroplast*'"):ti,ab,kw OR ("'knee replacement"):ti,ab,kw OR ("knee prosthes*"):ti,ab,kw | 2,388 |
| #6 | #3 OR #4 OR #5 | 3,689 |
| #7 | (unicompartmental):ti OR (unicondylar):ti | 112 |
| #8 | #6 AND #7 | 76 |
| #9 | (Lateral):ti,ab,kw OR (laterally):ti,ab,kw OR (medial):ti,ab,kw OR (medially):ti,ab,kw | 40,829 |
| #10 | MeSH descriptor: [Treatment Outcome] explode all trees | 127,565 |
| #11 | #10 OR #11 | 378,525 |
| #12 | #1 OR #2 OR #8 | 139 |
| #13 | #13 AND #9 AND #12 | 35 |
